# High AUROC can mask decision failure in sepsis transcriptomic classifiers: Preprocessing stability outweighs post hoc calibration across cohorts

**DOI:** 10.1371/journal.pone.0357585

**Published:** 2026-09-03

**Authors:** Hongwei Zheng, Wenbiao Chen

**Affiliations:** Quanzhou Medical College, Quanzhou, Fujian, People’s Republic of China; Sreenidhi Institute of Science and Technology, INDIA

## Abstract

**Background:**

High AUROC is often taken as evidence that a transcriptomic classifier is promising, but rank discrimination can conceal fixed-threshold failure after cohort or platform transfer.

**Methods:**

We benchmarked four GEO whole-blood cohorts: GSE65682 for discovery, GSE95233 for external microarray validation, GSE154918 for cross-platform RNA-seq validation, and GSE28750 for non-infectious inflammation stress testing. We compared logistic-regression workflows using training-derived standard scaling, training-derived robust scaling, sample-wise rank normalization with training-derived scaling, and robust scaling using unsupervised external-cohort reference statistics. Internal performance used five-fold cross-validation with fold-contained imputation and scaling. External uncertainty used 2,000 stratified bootstrap replicates.

**Results:**

Internal discrimination was very high for all strategies, but external validation revealed threshold collapse for training-derived standard and robust scaling. In GSE154918, both had balanced accuracy 0.50 at the 0.5 threshold despite very high AUROC, equivalent to random classification at that fixed threshold. The strict-inductive sample-rank strategy preserved fixed-threshold performance across external cohorts (balanced accuracy 0.95–1.00). Robust external-cohort adaptation also performed well (0.95–1.00) but uses unlabeled external-cohort distribution statistics and is therefore reported as adaptation rather than fixed single-sample transfer. Calibration and regularization sensitivity did not rescue the failing training-derived scaling strategies. In the sepsis-versus-non-infectious-inflammation stress test, robust external-cohort adaptation had the highest observed balanced accuracy (0.80, 95% CI 0.61–0.95), but its difference from sample-rank normalization was uncertain in paired bootstrap analysis.

**Conclusions:**

High AUROC can mask fixed-threshold failure in sepsis transcriptomic classifiers. In this benchmark, strict-inductive sample-rank normalization was the most stable fixed external strategy, while robust external-cohort scaling was best interpreted as unsupervised cohort adaptation whose reliability depends on external reference-sample availability.

## Introduction

Sepsis is defined as life-threatening organ dysfunction caused by a dysregulated host response to infection and remains a major global cause of death and critical illness [1,2]. Because early recognition remains difficult, whole-blood host-response profiling has attracted sustained interest as a route to diagnosis-oriented and stratification-oriented research [3]. At the same time, biomarker implementation in sepsis has remained difficult, in part because cohort heterogeneity, comparator choice, and evaluation design can all distort apparent performance [3].

One recurring problem is that transcriptomic models are often developed under analytically permissive conditions. Early molecular diagnostic work showed that host-response assays can separate sepsis-related states under defined development and validation settings [4]. More recent public-data machine-learning studies have also used compact signatures and internal or discovery-stage validation to nominate sepsis biomarkers or prognostic models [5,6]. Those designs may still be useful for hypothesis generation, but they do not answer the more practical question of whether a fixed workflow remains numerically stable after cohort shift, platform shift, or replacement of healthy controls with harder inflammatory comparators.

This distinction matters because rank discrimination and threshold behavior are not the same property. A workflow may preserve AUROC while still failing at the level where a classification threshold would be applied. In that setting, strong internal discrimination and strong external ordering can coexist with an unstable external score scale. From a translational perspective, that hidden decision failure is more important than another increment in internal cross-validation performance because externally transferred models are interpreted at the level of fixed scores and thresholds, not at the level of retrospective ranking alone.

Multicentre host-response studies with explicit validation demonstrate that blood RNA measurements can carry reproducible infection-related signal [7,8]. The unresolved gap is numerical transportability. Sepsis biomarker guidance emphasizes the difficulty of moving promising markers into robust implementation [3]. In parallel, public transcriptomic machine-learning studies often prioritize biomarker nomination or prognostic modeling rather than direct testing of whether fixed scores and thresholds remain usable after transfer [5,6]. Biomarker discovery and workflow evaluation are therefore often conflated.

We therefore interrogated the numerical transportability of classification thresholds rather than proposing another sepsis signature. Using a multi-cohort whole-blood GEO benchmark, we tested whether AUROC can overstate classifier usefulness once evaluation moves from internal validation to fixed external transfer. We intentionally used conservative phenotype harmonization, gene-level feature alignment, simple baseline models, and fixed external evaluation so that transportability failures would remain interpretable rather than being obscured by modeling flexibility.

The core hypothesis was that externally stable transcriptomic classification depends more on decision-score stability under transfer than on nominal internal discrimination, and that preprocessing rigor matters more than post hoc calibration or nominal model complexity for preserving that stability. We also expected that workflow ranking would change once the benchmark moved from biologically distant healthy controls to harder inflammatory negative comparators. This design follows the logic of transparent prediction-model reporting, where the credibility of a model study depends not only on discrimination but on how clearly the development and evaluation process has been made visible [9,10].

## Materials and methods

### Study design

**Fig 1:**
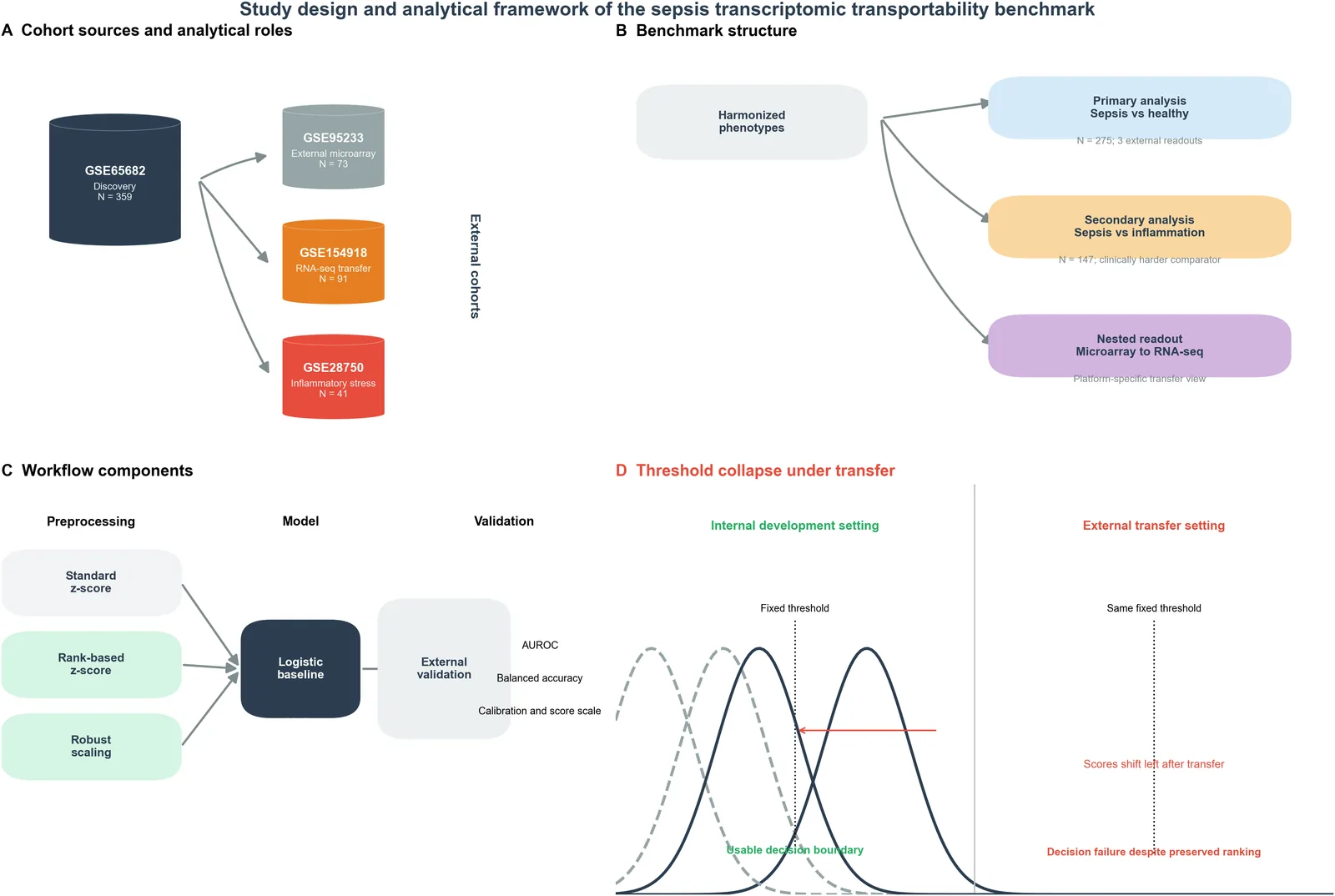
Study design and analytical framework of the transportability benchmark. Panel A summarizes the four public whole-blood cohorts and their assigned analytical roles: GSE65682 for discovery, GSE95233 for external microarray validation, GSE154918 for RNA-seq transfer validation, and GSE28750 for inflammatory-comparator stress testing. Panel B outlines the benchmark structure, including the primary sepsis-or-shock-versus-healthy analysis (n = 275), the sepsis-versus-non-infectious-inflammation analysis (n = 147), and the nested RNA-seq transfer readout. Panel C summarizes the workflow comparison from preprocessing to fixed external validation and decision-focused metrics. Panel D illustrates the interpretive framework: high AUROC can coexist with fixed-threshold failure after transfer when score scale is not preserved.

This study evaluated how phenotype harmonization, preprocessing stability, and validation design influence the transportability of whole-blood transcriptomic classifiers for sepsis-related tasks using public GEO cohorts. The emphasis was on robustness of an existing workflow under external transfer rather than de novo signature discovery [9,10]. That emphasis is aligned with guidance that prediction studies should report discrimination and calibration together rather than rely on AUROC alone [11,12]. Decision-curve and risk-of-bias frameworks further motivate separating threshold behavior, applicability, and model-evaluation context from headline discrimination metrics [13,14]. A study-design overview is provided in Fig 1.

All analyses were restricted to processed public expression matrices. This was done deliberately to keep the workflow reproducible on modest local compute and to avoid raw-read reprocessing as an additional untracked source of technical heterogeneity. Study size was determined by availability of eligible public cohorts rather than by a prospective sample-size calculation. We therefore treated this as a convenience benchmark built from all public cohorts that met the eligibility criteria defined for this retrospective benchmark.

### Public cohorts and analytical roles

Four public whole-blood cohorts were assigned analytical roles before model benchmarking: GSE65682 as the primary discovery cohort, GSE95233 as the external microarray validation cohort, GSE154918 as the RNA-seq transfer validation cohort, and GSE28750 as the non-infectious inflammation stress-test cohort. Cohorts were included only if processed matrices were publicly available and their metadata supported conservative harmonization into phenotype label groups. A summary of cohort roles, retained labels, included and excluded samples, and analysis membership is provided in Table 1.

**Table 1:** Cohort roles, phenotype-harmonization counts, and analysis membership for the public whole-blood benchmark cohorts. The table summarizes GEO accession, platform type, total series size, samples retained after conservative phenotype harmonization, excluded samples, labels available after harmonization, assigned analytical role, task membership, and gene-level feature counts. Retained counts describe samples kept in the unified metadata table after phenotype harmonization and expression-matrix availability checks; task-membership counts describe the smaller binary-contrast subsets used for each benchmark analysis. Feature counts refer to gene-level rows retained after platform-specific probe or gene-symbol harmonization.

Cohort	Platform	Retained	Excluded	Benchmark Role	Analysis Membership
GSE65682	Microarray	359	443	Primary discovery cohort	Primary analysis; Inflammatory comparator analysis; Cross-platform readout
GSE95233	Microarray	73	51	External microarray validation	Primary analysis; Cross-platform readout
GSE154918	RNA-seq	91	14	Cross-platform RNA-seq validation	Primary analysis; Cross-platform readout
GSE28750	Microarray	41	0	Inflammatory stress-test cohort	Primary analysis; Inflammatory comparator analysis; Cross-platform readout

After phenotype harmonization and first-pass inclusion filtering, GSE65682 contributed 359 analyzable samples to the unified metadata table, GSE95233 contributed 73, GSE154918 contributed 91, and GSE28750 contributed 41. The larger retained count for GSE65682 reflects all harmonized labels available for the unified metadata table; only the subset matching each binary contrast entered a given benchmark task. Thus, 93 GSE65682 samples entered the primary sepsis-or-shock-versus-healthy training analysis, whereas infection-but-not-sepsis and non-infectious-inflammation samples were retained for metadata traceability or for the secondary comparator analysis. Several source cohorts were assembled before Sepsis-3 and do not support retrospective relabeling to a single modern consensus definition from processed GEO records alone. We therefore harmonized labels against the original study descriptions and recoverable processed metadata, and we report GEO accessions, platform types, and benchmark roles rather than incomplete cohort-date fields.

To distinguish genuinely unavailable variables from merely unmodeled ones, we also performed a cohort-level metadata completeness audit across age, sex, infection or clinical context, severity or outcome, timepoint or follow-up, and treatment or exposure fields (S3 Table in S1 File).

### Phenotype harmonization

Phenotype harmonization was intentionally conservative. Samples were mapped into a compact label dictionary consisting of healthy, infection_non_sepsis, sepsis, septic_shock, and noninfectious_inflammation. Samples lacking defensible metadata support for this mapping were excluded rather than forced into pooled labels. For GSE95233, only healthy controls and day-1 septic-shock samples were retained, while later follow-up septic-shock samples were excluded. For GSE154918, Hlty, Inf1_P, Seps_P, and Shock_P samples were retained, whereas follow-up samples were excluded. For GSE28750, all healthy, sepsis, and post-surgical samples were retained. For GSE65682, only healthy subjects and ICU samples with metadata signals consistent with abdo_s, ctrl_GI, cap, hap, or no-cap were retained for the present benchmark.

This strategy prioritized label defensibility over maximal sample count because the benchmark objective was workflow stability under realistic but interpretable phenotype definitions. The main risk of this decision is reduced sample size; the benefit is a cleaner comparator structure for external failure-mode analysis.

### Expression matrix extraction and gene-level harmonization

Per-cohort included-sample expression matrices were extracted from GEO series matrices or, where required, from GEO supplementary processed files. Array-based cohorts were then collapsed to the gene-symbol level using GEO platform annotation tables. Probe rows were retained only if the platform Gene Symbol field mapped conservatively to a single symbol. Probe rows with blank annotation or multiple mapped symbols were excluded. When multiple probe rows mapped to the same gene, they were collapsed using the cohort-level median rather than selecting the highest-variance or best-performing probe.

RNA-seq data from GSE154918 were harmonized at the gene-symbol level from processed matrix annotation columns and were collapsed by the same median rule when duplicate gene symbols were present. This produced gene-level matrices for all four cohorts. The resulting harmonization statistics were as follows: GSE154918 retained 19,203 gene rows; GSE28750 and GSE95233 retained 21,815 gene rows each; and GSE65682 retained 11,222 gene rows. The smaller GSE65682 feature space reflects conservative single-symbol probe mapping rather than downstream model-stage data loss. Shared gene spaces were defined at the task level by intersecting retained gene symbols before model fitting. This operation used feature availability only and did not use outcomes, predicted scores, external-cohort labels, or external model performance. The gene intersection was therefore fixed for each task before internal cross-validation began rather than reselected inside each fold.

### Predefined benchmark analyses

Two benchmark analyses were defined before modeling, with cross-platform transfer treated as a predefined property of the primary analysis rather than as a separate discovery branch.

The primary analysis evaluated sepsis or septic shock versus healthy controls across four cohorts. This analysis contained 275 samples and 9,508 shared genes. Within it, GSE65682 contributed 93 training samples, GSE95233 contributed 73 external-validation samples, GSE154918 contributed 79 RNA-seq transfer-validation samples, and GSE28750 contributed 30 additional external stress-test samples. The GSE154918 subset was interpreted as the cross-platform robustness readout for the primary analysis.

The secondary analysis evaluated sepsis versus noninfectious_inflammation. This analysis contained 147 samples and 9,984 shared genes. GSE65682 served as the discovery cohort with 126 samples, and GSE28750 served as the external stress-test cohort with 21 samples.

The cohort roles, primary contrast, secondary contrast, and RNA-seq transfer readout were defined before model fitting. Threshold sweeps, paired bootstrap differences, calibration diagnostics, regularization sensitivity, coefficient-stability summaries, and adaptive-reference-size sensitivity were added during revision as diagnostic or sensitivity analyses to clarify the observed failure modes.

### Workflow factors under comparison

The first benchmarking stage focused on preprocessing and external-reference strategy rather than on proliferation of model families. Four strategies were compared so that strictly inductive transfer and unsupervised external-cohort adaptation were separated explicitly.

Standard scaling (train-only) used per-feature median imputation followed by feature-wise standard scaling. For internal cross-validation, the imputer and scaler were fitted within each training fold and applied to the held-out fold. For external evaluation, the imputer and scaler were fitted once on the full GSE65682 training cohort and applied unchanged to each external cohort.

Robust scaling (train-only) used per-feature median imputation followed by median and interquartile-range scaling with all reference statistics estimated from the training fold or training cohort only. This strategy represents the strict-inductive counterpart to robust scaling.

Sample-wise rank normalization first replaced each sample’s expression values with within-sample percentile ranks and then applied feature-wise standard scaling with training-derived parameters. Because the rank step is sample-wise, external transformation does not require information from other external samples.

Adaptive robust scaling (external reference) used the same training-cohort robust scaling for model fitting, but external samples were transformed using medians and interquartile ranges estimated from the unlabeled external cohort. This strategy does not use external class labels, but it does use the external-cohort feature distribution. We therefore treat it as unsupervised external-cohort adaptation or transductive preprocessing, not as a strictly inductive fixed-model workflow for individual external samples scored one at a time.

These strategies encode different assumptions about how an external cohort should be aligned to the score axis learned in the training cohort. Standard z-score preprocessing anchors each feature to the training-cohort mean and standard deviation. Training-only robust scaling changes the reference estimator but remains fixed after training. Sample-wise rank normalization reduces dependence on absolute measurement scale by replacing each sample with within-sample order statistics before feature scaling. Robust external-cohort adaptation uses unlabeled external-distribution information to estimate a new reference frame. The comparison was motivated by foundational work showing that batch effects can dominate high-throughput expression analyses [15,16]. Cross-study normalization and platform-reproducibility studies further show that expression measurements can require explicit alignment before model transfer [17–19]. The external-adaptation arm was also informed by clinical dataset-shift work, where robustness depends on how distributional change is handled at evaluation time [20].

### Baseline model and full model specification

The baseline model family was logistic regression with L2 regularization, C = 1.0, class_weight = “balanced,” solver = “liblinear,” max_iter = 5000, and fixed random seed 20260313. The regularization setting was the scikit-learn default for this solver and was not selected by nested cross-validation. This model was chosen deliberately because the benchmark question was whether preprocessing and validation design alter transportability, not whether a higher-capacity learner can produce marginally better internal performance. A simple linear model provides a cleaner probe of score-scale behavior than a nonlinear learner that may entangle preprocessing effects with model flexibility.

To improve reproducibility, fixed intercepts and coefficient tables for all fitted training-cohort baselines were exported after model fitting on the full GSE65682 training set and are summarized in S2 Table in S1 File. We also exported fold-level coefficient summaries and pairwise fold-stability diagnostics (S10 Table in S1 File) and repeated the external benchmarks across multiple L2 regularization strengths (C = 0.01, 0.03, 0.1, 0.3, 1, 3, 10; S9 Table in S1 File).

### Internal and external validation design

The primary development cohort was GSE65682. Internal performance was estimated by five-fold stratified cross-validation within that cohort. In each fold, median imputation, scaling, and model fitting were learned only from the fold-training subset and then applied to the held-out fold. External performance was evaluated by training each strategy once on the full GSE65682 training cohort and then scoring held-out cohorts without label-based retuning. For the three strict-inductive strategies, all preprocessing parameters used for external scoring were training-derived or sample-wise. For adaptive robust scaling, external medians and interquartile ranges were estimated from the unlabeled external feature matrix and are reported separately as adaptation. No external labels were used for fitting, threshold selection, calibration, or regularization selection.

For each strategy, internal cross-validation predictions were also used to derive an empirical training-cohort optimal threshold based on balanced accuracy. External datasets were then evaluated at both the default 0.5 threshold and the training-derived threshold. The 0.5 threshold is not claimed to be application-optimized, particularly because class weights and prevalence differ across cohorts. It is used here as a fixed numerical reference to expose score-scale displacement. Threshold sweeps, sensitivity, specificity, positive predictive value, negative predictive value, confusion matrices, observed positive rates, and predicted positive rates are provided in S4 Table and S5 Table in S1 File.

### Calibration benchmark

A secondary benchmark tested whether post hoc probability calibration could recover external fixed-threshold performance once preprocessing had already shifted score scale. Calibration was evaluated with three settings: no calibration, sigmoid calibration, and isotonic calibration. Calibrated models used five-fold internal cross-validation within GSE65682; calibration models were fit from training-cohort data only, and external cohorts remained untouched during calibration. The purpose of this analysis was not to optimize probabilities in isolation but to determine whether calibration could compensate for unstable preprocessing under cross-cohort transfer. Calibration intercepts, slopes, expected calibration error, and curve source data are reported in S7 Table in S1 File and the archived calibration source file.

### Performance metrics and uncertainty estimation

Performance was evaluated with AUROC, average precision, balanced accuracy, Brier score, predicted positive rate, and class-specific median predicted scores. AUROC and average precision were used to summarize ranking performance. Balanced accuracy was emphasized because the benchmark focused on threshold usability under class imbalance and cohort shift. Brier score was used as a coarse measure of probability quality. Predicted positive rate was compared with observed positive rate to diagnose threshold collapse and score-scale drift. This metric set was chosen to keep ranking, calibration, and threshold-level utility analytically separate. Calibration and traditional performance summaries were interpreted according to prediction-model evaluation guidance [11,12]. Threshold-level behavior was considered separately because decision consequences and class-imbalance corrections can diverge from rank discrimination [13,21]. We also considered individual-level prediction stability and threshold-based sample-size considerations when interpreting external decision performance [22,23].

For the key external analyses, 95% confidence intervals were estimated with 2,000 stratified bootstrap replicates that resampled positive and negative classes separately within each external cohort. These are percentile intervals. Resampling was class-stratified, so each bootstrap replicate retained both positive and negative classes; this avoided degenerate one-class AUROC and balanced-accuracy replicates. Separate uncertainty summaries are provided in S1 Table in S1 File, and paired stratified bootstrap differences between workflows are provided in S6 Table in S1 File.

### Bias handling, missing data, and ethics

Several design choices were intended to reduce optimism and leakage. Cohort roles were fixed before modeling, phenotype harmonization was conservative, gene-space intersections were defined separately for each analysis but remained deterministic, and external evaluation used fixed labels only for evaluation. Missing expression values were handled by per-feature median imputation inside each training fold or training cohort. The processed gene-level matrices used in the benchmark contained no missing numeric expression entries after matrix extraction, so the imputation step functioned mainly as a fold-contained safeguard rather than as a major source of variation. We did not use KNN or iterative imputation because no expression-missingness pattern required model-based reconstruction and because such methods would have added extra tuning choices to a preprocessing-focused benchmark. Treatment variables and demographic fairness analyses were not modeled because those fields were not consistently recoverable across processed public GEO records; this is treated as a reporting and generalizability limitation rather than ignored silently.

The study used publicly available, de-identified human data only and did not involve new participant recruitment, contact, or access to identifiable records. Ethics approval and consent were addressed in the original source studies. No additional institutional ethics approval was required for this secondary analysis of public datasets.

### Software and reproducibility

All data extraction, harmonization, task building, benchmarking, figure generation, confidence-interval estimation, and coefficient export were implemented in a reproducible analysis workflow. The revised analysis was run with Python 3.9.6, NumPy 1.26.4, pandas 2.3.2, scikit-learn 1.6.1, matplotlib 3.9.4, seaborn 0.13.2, and requests 2.32.5. The corresponding environment file is requirements.txt, and the analysis code is released under the MIT License. GEO series matrices, supplementary processed files, and platform annotations were downloaded locally on 2026-03-13; source URLs and local file records are retained in data/manifest.tsv. The revision workflow can be rerun with scripts/reproduce_one_click.sh, which regenerates task datasets, benchmarks, diagnostics, figures, and supporting tables. The pipeline used processed public matrices and CPU-friendly models and can be rerun in a standard Python environment on consumer-grade workstations. This constraint was intentional because the manuscript argues for reproducible and accessible public-data benchmarking rather than for compute-intensive bespoke pipelines.

## Results

### Cohort assembly and benchmark structure

**Fig 2:**
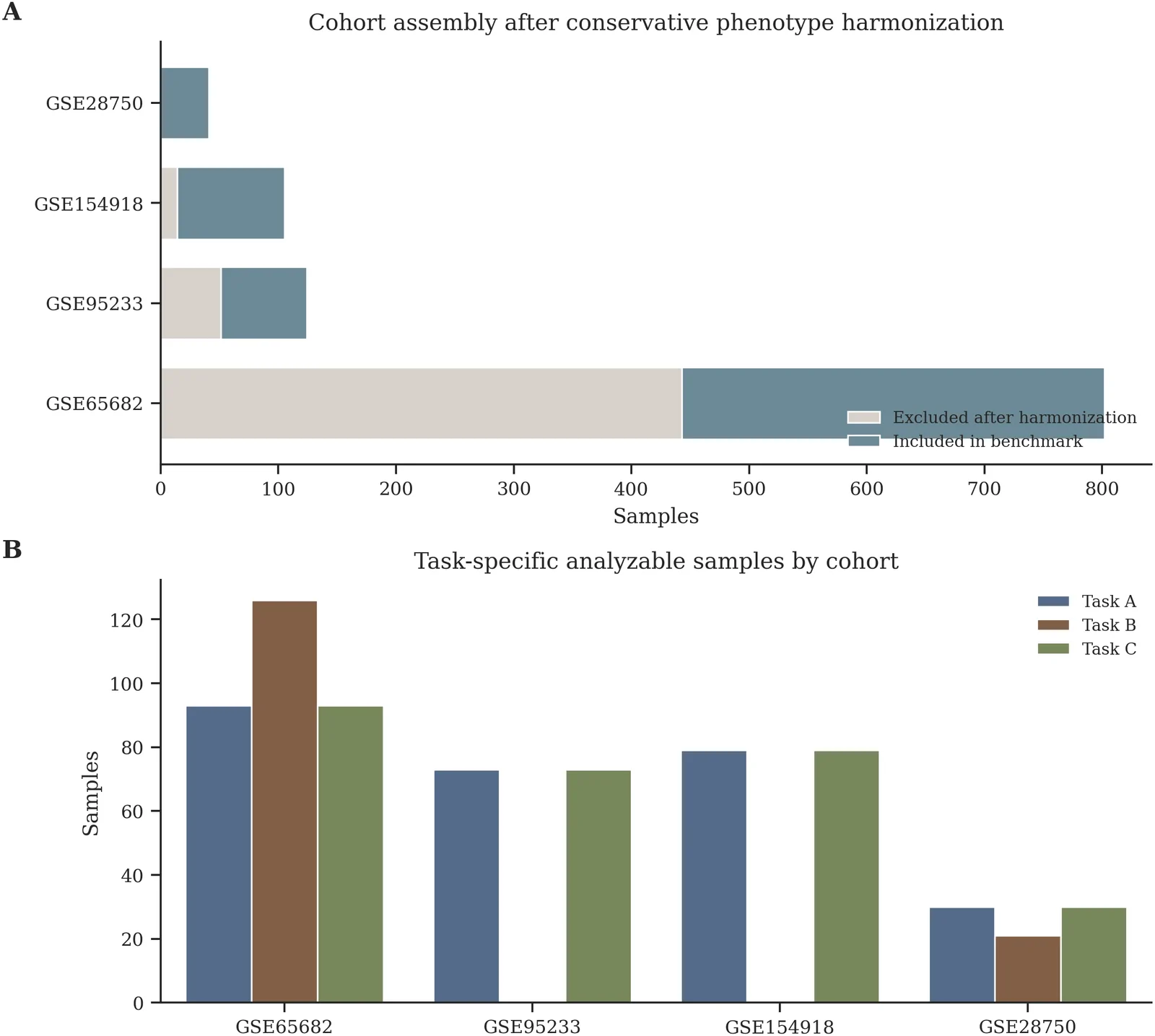
Cohort-flow summary for the benchmark dataset assembly. The diagram shows how four public whole-blood GEO cohorts were screened, harmonized, and assigned to benchmark roles. It reports the total series size, exclusions applied during conservative phenotype harmonization, mainline included samples, and cohort-specific samples entering the primary sepsis-or-shock-versus-healthy analysis, the sepsis-versus-non-infectious-inflammation analysis, and the nested RNA-seq transfer readout. Counts are sample counts after phenotype harmonization and expression-matrix availability checks.

The benchmark assembled four whole-blood public cohorts with conservative phenotype mappings and fixed analytical roles. Table 1 summarizes cohort roles, included and excluded samples, and analysis membership, while Fig 2 provides the cohort-flow overview. The difference between the 359 retained GSE65682 metadata samples and the 93 primary-training samples reflects task membership: only healthy and sepsis/septic-shock samples entered the primary contrast, while other retained labels supported metadata accounting or the secondary comparator analysis. After phenotype harmonization and feature-level filtering, the primary sepsis-or-shock-versus-healthy analysis contained 275 analyzable samples across four cohorts with 9,508 shared genes, including a predefined RNA-seq transfer readout in GSE154918. The secondary sepsis-versus-noninfectious-inflammation analysis contained 147 samples across two cohorts with 9,984 shared genes. This design allowed us to evaluate not only within-cohort discrimination but also threshold stability under external, cross-platform, and harder-negative settings.

Within the primary development cohort (GSE65682), a simple logistic-regression baseline achieved essentially perfect internal discrimination regardless of preprocessing strategy: internal cross-validation balanced accuracy and AUROC were both 1.00 for all four preprocessing strategies (S4 Table in S1 File). In the secondary sepsis-versus-non-infectious-inflammation analysis, internal AUROC was 0.948–0.950 and internal balanced accuracy at the default threshold was 0.904–0.918, depending on preprocessing strategy (S4 Table in S1 File). At the level of internal cross-validation alone, every workflow appeared successful. Taken in isolation, these results would support an overly optimistic reading of public-data biomarker performance.

### Diagnostic threshold instability under external transfer

When the fixed workflow was transferred outside the discovery cohort, training-derived standard z-score preprocessing retained very high AUROC across GSE95233, GSE154918, and GSE28750, but the default 0.5 threshold assigned all external samples to one side of the decision boundary, yielding balanced accuracy of 0.50 in all three external cohorts. The corresponding bootstrap confidence intervals for balanced accuracy were degenerate at 0.50–0.50, whereas AUROC remained 1.00 in GSE95233, 0.999 (95% CI 0.994–1.000) in GSE154918, and 1.00 in GSE28750 (S1 Table in S1 File). Training-derived robust scaling did not solve this fixed-transfer problem: balanced accuracy was 0.57 in GSE95233, 0.50 in GSE154918, and 0.55 in GSE28750.

**Fig 3:**
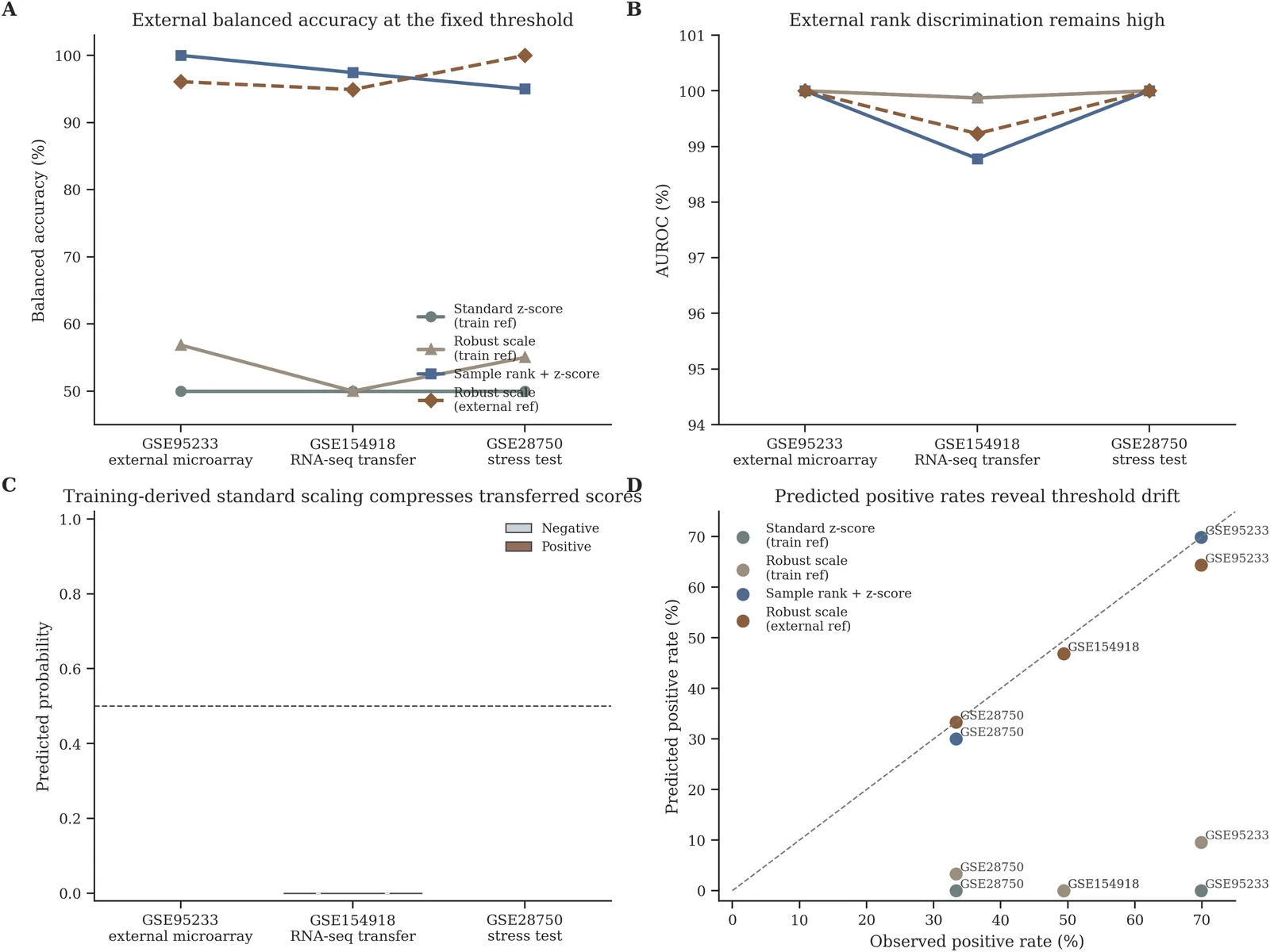
External threshold failure depends primarily on preprocessing and reference strategy. The figure summarizes the primary sepsis-or-shock-versus-healthy external evaluations after training on GSE65682 (n = 93; 9,508 shared genes). External cohorts were GSE95233 (n = 73), GSE154918 (n = 79), and GSE28750 (n = 30). Panel A reports external balanced accuracy at the fixed 0.5 threshold for training-only standard scaling, training-only robust scaling, sample-wise rank normalization, and robust external-cohort adaptation. Panel B reports the corresponding AUROC values, showing that discrimination alone obscures the failure of training-derived feature scaling. Panel C displays uncalibrated transferred logistic-regression probabilities for the standard z-score train-only workflow across the three external cohorts. Panel D compares observed and predicted positive rates, showing that threshold collapse arises because transferred scores sit below the fixed decision axis rather than because class ranking has disappeared.

This was the central failure mode of the benchmark. Ranking survived, but the fixed numerical threshold no longer separated classes after transfer. For the standard z-score train-only workflow, the predicted positive rate was 0.0 in each primary external cohort at the 0.5 threshold, meaning that no external sample was assigned to the positive class despite preserved AUROC (S4 Table in S1 File). A reader focusing only on AUROC would conclude that the external model was excellent, whereas a reader examining fixed-threshold classification would conclude that the same model did not function as a thresholded classifier. The problem was therefore not generic poor calibration in the abstract and not complete loss of biological signal. It was consistent with score-scale displacement after transfer (Fig 3).

### Strict-inductive and adaptive strategies separated the main finding

Under a strictly inductive design, sample-wise rank normalization followed by training-derived z-score scaling was the most stable external strategy. It achieved balanced accuracy of 1.00 in GSE95233, 0.974 in GSE154918, and 0.95 in GSE28750, with predicted positive rates much closer to observed positive rates than the two training-derived scaling strategies. Robust external-cohort adaptation also performed well (0.961, 0.949, and 1.00, respectively), but this strategy used unlabeled external-cohort medians and interquartile ranges. We therefore interpret it as an adaptive cohort-level normalization option rather than as a fixed single-sample transfer pipeline.

The same point held when the primary external matrix was read explicitly as a cross-platform transfer problem (S1 Fig). In GSE154918, the two training-derived feature-scaling strategies preserved ranking but assigned no external samples above the fixed 0.5 threshold. In contrast, sample-wise rank normalization and adaptive robust scaling preserved high fixed-threshold performance. The platform-transfer result broadens the main conclusion; it does not create a second discovery claim.

### Post hoc calibration did not rescue a poor preprocessing choice

We next tested whether post hoc probability calibration could repair external threshold failure once preprocessing had already distorted score scale. For standard scaling (train-only) and robust scaling (train-only), adding sigmoid or isotonic calibration did not recover fixed-threshold performance in the external cohorts where all predicted probabilities remained below the decision threshold. By contrast, calibration applied after sample-wise rank normalization or adaptive robust scaling produced only modest changes relative to the already strong uncalibrated results.

The practical improvement came from restoring a transferable score scale rather than from extra post hoc tuning. Calibration may refine probabilities when the underlying score scale is already coherent, but it does not compensate for preprocessing choices that place external cohorts on the wrong numerical scale.

Consistent with that interpretation, the fitted coefficient vectors for the primary analysis remained highly concordant across preprocessing workflows despite their different external threshold behavior (S2 Fig). Pairwise Pearson correlations ranged from 0.938 to 0.969, Spearman correlations from 0.946 to 0.976, and coefficient-sign agreement from 0.909 to 0.951. Fold-level coefficient correlations were also high in the primary analysis (mean Pearson correlations approximately 0.94 across preprocessing methods; S10 Table in S1 File). Recurrent high-weight genes shown in S2 Fig, including RNASE1, PTGFR, ZNF608, UBXN2A, HTRA1, and KIAA1324, provide a qualitative check that the fitted models were not arbitrary coefficient vectors. We do not interpret these genes as a validated biomarker panel or as pathway-enrichment evidence, because the study was not designed for mechanistic feature discovery and correlated transcriptomic predictors can share coefficient structure without establishing individual-gene causality. The coefficient summaries nevertheless show that the failing strategies did not simply learn an unrelated linear signal.

### Inflammatory comparators altered workflow ranking in a small stress test

The secondary analysis tested sepsis against non-infectious inflammation using GSE65682 as the discovery cohort and GSE28750 as an external stress test. As expected, this was a harder problem than sepsis-versus-healthy discrimination. Internal cross-validation AUROC dropped from approximately 1.00 in the primary analysis to approximately 0.95 in the secondary analysis, indicating substantially greater overlap between positive and negative classes.

The preprocessing hierarchy also changed in this harder setting. Standard z-score preprocessing and training-derived robust scaling both assigned all 21 external samples below the 0.5 threshold, giving balanced accuracy of 0.50 despite AUROC values of 0.873 and 0.836, respectively. The rank-based workflow improved external balanced accuracy to 0.70 (95% CI 0.55–0.85), with 4 true positives, 11 true negatives, 0 false positives, and 6 false negatives. Robust external-cohort adaptation had the highest observed balanced accuracy (0.805, 95% CI 0.614–0.950) and the lowest Brier score (0.158), with 7 true positives, 10 true negatives, 1 false positive, and 3 false negatives (S1 Table, S4 Table in S1 File).

**Fig 4:**
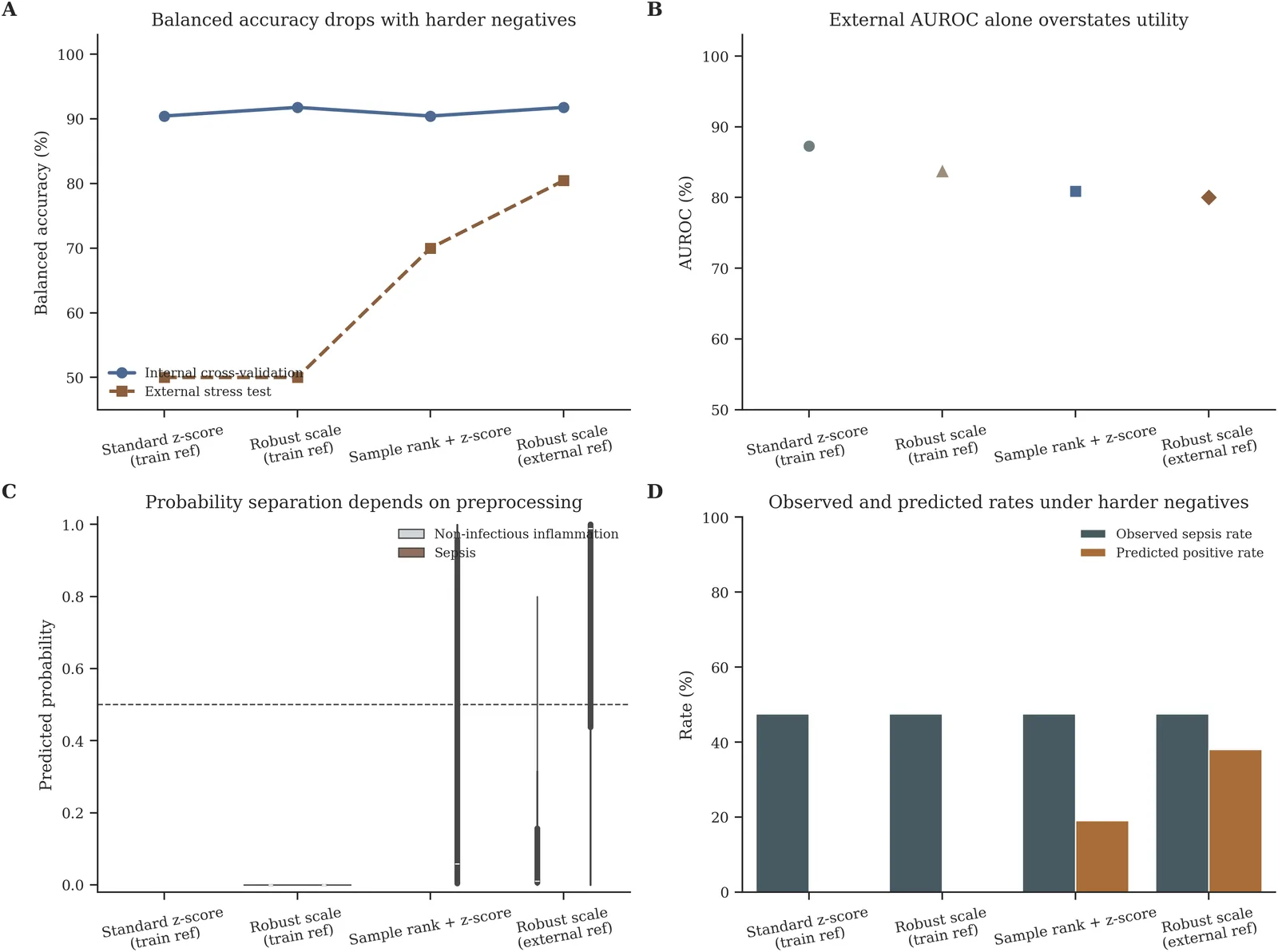
Inflammatory comparators alter workflow ranking and reveal limits beyond score-scale drift. This figure summarizes the sepsis-versus-non-infectious-inflammation analysis using GSE65682 as the development cohort (n = 126; 9,984 shared genes) and GSE28750 as the external stress-test cohort (n = 21; 10 sepsis and 11 non-infectious-inflammation samples). Panel A compares internal and external balanced accuracy across strict-inductive and adaptive preprocessing strategies. Panel B shows that AUROC can still overstate fixed-threshold performance under an inflammation-confounded negative class. Panel C displays external score distributions in the stress-test cohort for each strategy. Panel D contrasts observed and predicted positive rates and highlights that robust external-cohort adaptation had the highest observed fixed-threshold performance in this small external cohort. Because the paired bootstrap interval versus sample-wise rank normalization crossed zero, this panel is interpreted as exploratory rather than as definitive workflow ranking.

Because this external stress test contained only 10 sepsis and 11 non-infectious-inflammation samples, the apparent ranking between sample-wise rank normalization and adaptive robust scaling should be treated as exploratory. The paired bootstrap difference in balanced accuracy for robust external adaptation versus sample-rank normalization was 0.105, with a 95% percentile interval from −0.045 to 0.255 (S6 Table in S1 File). These findings suggest that once the negative class becomes inflammation-confounded, preserving a transferable score scale is still necessary but no longer sufficient; however, this small stress-test cohort does not support a definitive ranking of preprocessing strategies (Fig 4).

### Archived fixed baseline models support reproducibility

To reduce ambiguity about what exactly was evaluated, full-model intercepts and coefficient tables were exported for all training-cohort logistic baselines and summarized in S2 Table in S1 File. The fitted intercepts were close to zero, consistent with the use of class_weight = “balanced” and with a decision boundary that depends strongly on the scaled gene-expression inputs. This is why a preserved coefficient vector can still fail at a fixed threshold if preprocessing moves the external samples onto a different score scale. Regularization-strength sensitivity showed that the external fixed-threshold pattern was not removed by changing L2 C across the tested grid (S9 Table in S1 File). We also evaluated the external-reference size required for robust adaptation as a revision-added sensitivity analysis. Small reference subsets were more variable: for example, across the 100 5-sample adaptive-reference draws for GSE154918 in S8 Table in S1 File, mean balanced accuracy was 0.897 with minimum 0.679, whereas the full 79-sample reference produced 0.949. This supports treating robust external-cohort scaling as a batch or cohort adaptation method whose reliability depends on reference-sample availability.

## Discussion

The central implication of this benchmark is straightforward: in public whole-blood sepsis transcriptome studies, transportability depends less on headline internal discrimination than on whether preprocessing preserves a stable numerical score scale after transfer. The revised analysis changes the original interpretation in an important way. Training-derived robust scaling did not reproduce the performance of robust scaling that used external-cohort reference statistics. Under strict inductive transfer, sample-wise rank normalization was the most stable fixed external strategy. Robust external-cohort scaling remained useful, but it should be described as unsupervised cohort adaptation rather than as direct evidence of strict-inductive single-sample transfer.

External failure in this setting was not adequately described as generic poor calibration, nor as simple loss of discriminatory information. Ranking survived while the fixed threshold failed, which is consistent with score-scale instability under cohort shift. That interpretation remains bounded: the present design does not isolate whether platform, prevalence, case mix, sampling frame, or technical batch contributed most to the displacement. The practical point is narrower and still important: post hoc calibration did not rescue the external failure produced by unstable training-derived preprocessing. This pattern is consistent with prediction-model literature showing that calibration and global discrimination measure different properties [11,12]. It also aligns with decision-focused work showing that threshold behavior and class-imbalance handling can diverge from headline discrimination metrics [13,21]. Individual-level prediction stability and threshold-based sample-size requirements further support treating fixed-threshold behavior as a separate evaluation target [22,23].

At the preprocessing level, one plausible explanation is that training-derived feature scaling remained sensitive to between-cohort shifts in feature medians, variances, and interquartile ranges. Such shifts can move external samples onto a different numerical score scale even when their relative ordering remains informative. Sample-wise rank normalization reduces this dependence on absolute expression scale by transforming each sample through within-sample order statistics before applying training-derived scaling. This interpretation is statistical rather than mechanistic: it helps explain the observed transport behavior but does not identify which biological, platform, or sampling component caused the displacement.

This reading also fits the broader sepsis biomarker landscape. Sepsis pathobiology and guideline literature underscore why cohort composition and comparator choice can vary substantially across care contexts [24,25]. Contemporary reviews emphasize methodological heterogeneity and persistent difficulty translating apparently promising sepsis biomarkers into practice [3,26]. Rigorous multicentre host-response studies have shown that blood transcriptional signals can support infection and sepsis stratification when validation is explicit and prospective or multicentre by design [7,8]. Prospective genomic landscape work similarly shows the value of externally visible cohort design when relating host response to sepsis outcomes [27]. A separate body of sepsis transcriptomic work has focused on mortality prediction and molecular subphenotypes [28,29]. Public-data diagnostic benchmarking and independent validation studies have provided important evidence on cross-study signal and comparator choice [30–32]. Compact host-response signatures have also been developed for bacterial/viral or respiratory infection discrimination [33,34]. Those strands are informative, but they should not be read as interchangeable evidence for the transportability of fixed preprocessing-and-threshold workflows.

There is also a workflow-governance implication. A fixed model object is not sufficient protection against transport failure if the preprocessing logic that generates its inputs is itself unstable across cohorts or platforms. Model weights and preprocessing should therefore be reported together as the evaluated prediction workflow.

The harder-negative benchmark strengthens that point while adding a second one. When the negative class changed from healthy controls to non-infectious inflammation, the task became harder even in internal validation. Standard z-score preprocessing and training-derived robust scaling again failed at the decision level. Robust external-cohort adaptation had the highest observed performance, but the external stress test contained only 21 samples and the paired bootstrap interval versus sample-rank normalization crossed zero. This result is better read as a reality check showing that workflow ranking can change under inflammatory confounding, not as definitive evidence that one adaptive strategy is superior.

These results also position the manuscript against a recognizable pattern in public-data bioinformatics. Recent sepsis studies have combined public transcriptomic resources with machine learning to nominate biomarkers or prognostic signatures [5,6]. Public-data diagnostic benchmarks have provided useful evidence for cross-study signal and comparator choice [30–32]. Host-response signature studies have further shown that compact assays can be informative under defined diagnostic settings [33,34]. Our concern is narrower: such designs are less informative about whether a fixed score scale and threshold remain stable after transfer. That concern is not unique to sepsis; early high-dimensional microarray prediction work warned that class-separation claims can be fragile without careful validation [35,36]. Independent resampling and validation analyses reinforced that optimistic signatures may not transport reliably when development choices are perturbed [37]. Our study was designed to answer a different question. It does not attempt to nominate a new definitive biomarker panel. Instead, it asks which workflow properties remain trustworthy when public-data models leave the cohort in which they were optimized.

The cross-platform analysis should be read in that bounded sense. Here it functions as a platform-specific view of the main external benchmark rather than as a separate discovery branch. Its added value is interpretive: in this dataset collection, RNA-seq transfer preserved ranking while exposing fixed-threshold instability for training-derived feature scaling. Recent large-scale sepsis omics studies illustrate the biological richness of host-response measurement [38]. Precision-sepsis reviews and neonatal diagnostic validation work point to the same operational lesson: biological signal is not the same as externally stable prediction behavior, and generalization still has to be demonstrated rather than assumed [39,40].

The benchmark also has important limitations. Public cohorts were assembled across heterogeneous care contexts and with uneven metadata completeness. The metadata audit (S3 Table in S1 File) showed that baseline fields were only partially recoverable and often not comparable across datasets, so a pooled covariate table would have introduced false precision rather than greater transparency. The inflammatory-comparator external stress test is small: the sepsis-versus-non-infectious-inflammation comparison rests on 21 external samples in GSE28750. The analysis was intentionally centered on simple logistic baselines to isolate preprocessing and transfer effects; alternative model families may behave differently, and established compact signatures such as the Sepsis MetaScore, FAIM3-to-PLAC8 ratio, or SeptiCyte-style host-response panels were not reimplemented as separate classifiers. That narrower scope keeps the benchmark focused on preprocessing transportability rather than on comparing all available sepsis signatures. The 11,222-gene discovery feature space reflects conservative single-symbol probe mapping and exclusion of ambiguous probes rather than inadvertent downstream data loss. Demographic and treatment metadata were not harmonizable across processed GEO records, so fairness and treatment-effect analyses remain outside the scope of this benchmark [10].

Within those bounds, the manuscript makes a specific methodological claim rather than a broad deployment claim. It does not propose definitive biomarker discovery or universal transportability across all sepsis populations. Instead, it shows that a reproducible public-data benchmark can identify workflow properties that are consistently associated with better external behavior, and that those properties are not the ones most commonly emphasized in generic signature papers. For this dataset collection, preserving a stable cross-cohort score scale mattered more than achieving another headline internal AUROC.

The immediate practical recommendation is therefore conservative. Future public-data sepsis transcriptome studies should treat preprocessing, threshold behavior, harder negative comparators, and fixed external stress testing as first-order design elements rather than as implementation details. At minimum, such studies should report whether externally transferred scores remain stable at stated thresholds, whether workflow ranking changes when healthy controls are replaced by inflammation-confounded comparators, and whether post hoc calibration genuinely adds value after preprocessing has been chosen.

## Conclusion

In public whole-blood sepsis transcriptome studies, high AUROC can mask fixed-threshold failure after cohort and platform transfer. Strict-inductive sample-rank normalization was the most stable fixed external strategy in this benchmark, whereas robust scaling with external-cohort reference statistics is better interpreted as unsupervised cohort adaptation. A conservative benchmark that prioritizes preprocessing, threshold behavior, uncertainty reporting, and external stress testing therefore provides more useful evidence than another internally optimized signature report.

## Supporting information

S1 Fig Cross-platform transfer to the RNA-seq cohort highlights the same scale problem from a platform-specific view. The figure evaluates the primary sepsis-or-shock-versus-healthy classifier trained on the microarray discovery cohort and transferred to the RNA-seq cohort GSE154918 (n = 79; 39 positive and 40 negative samples). Panel A compares balanced accuracy and AUROC at the fixed 0.5 threshold across strict-inductive and adaptive preprocessing strategies. Panel B shows uncalibrated logistic-regression predicted probabilities by class. Panel C compares class-specific median transferred scores. Panel D contrasts preprocessing with post hoc calibration, showing that calibration does not restore fixed-threshold performance when training-derived preprocessing places all external scores on the wrong side of the decision boundary.
(PNG)

S2 Fig Coefficient concordance contextualizes, but does not prove, signal stability. The figure compares full-discovery-cohort logistic-regression coefficients for the primary analysis (GSE65682; n = 93; 9,508 shared genes). Panels A to C compare coefficient values across preprocessing workflows and show that fitted linear signals remain highly concordant even when external fixed-threshold behavior differs markedly. Panel D shows genes that remained within the top-50 absolute coefficients across all three underlying preprocessing algorithms. These summaries support the interpretation that threshold failure was not caused by learning an entirely unrelated linear signal, while remaining cautious because correlated high-dimensional genes can produce similar coefficient profiles without proving biological stability.
(PNG)

S1 File Supplementary tables and source data workbook. The Excel workbook contains S1-S11 Tables, the source-data sheets for Fig 3, Fig 4, S1 Fig, and S2 Fig, the calibration-curve source data, the fold coefficient pairwise-stability source data, and the source table for Table 1. The README sheet lists the source file, description, row count, and column count for each workbook sheet.
(XLSX)
